# Blood pressure regulation IX: cerebral autoregulation under blood pressure challenges

**DOI:** 10.1007/s00421-013-2667-y

**Published:** 2013-06-05

**Authors:** Yu-Chieh Tzeng, Philip N. Ainslie

**Affiliations:** 1 Cardiovascular Systems Laboratory, Centre for Translational Physiology, University of Otago, 23A Mein Street, PO Box 7343, Wellington South, New Zealand; 2 Centre for Heart, Lung and Vascular Health, School of Health and Exercise Sciences, University of British Columbia, Okanagan Campus, Vancouver, BC Canada

**Keywords:** Blood pressure, Blood pressure variability, Cerebral blood flow, Cerebral autoregulation, Circulation, Haemodynamics, Arterial

## Abstract

Cerebral autoregulation (CA) is integral to the delicate process of maintaining stable cerebral perfusion and brain tissue oxygenation against changes in arterial blood pressure. The last four decades has seen dramatic advances in understanding CA physiology, and the role that CA might play in the causation and progression of disease processes that affect the cerebral circulation such as stroke. However, the translation of these basic scientific advances into clinical practice has been limited by the maintenance of old constructs and because there are persistent gaps in our understanding of how this vital vascular mechanism should be quantified. In this review, we re-evaluate relevant studies that challenge established paradigms about how the cerebral perfusion pressure and blood flow are related. In the context of blood pressure being a major haemodynamic challenge to the cerebral circulation, we conclude that: (1) the physiological properties of CA remain inconclusive, (2) many extant methods for CA characterisation are based on simplistic assumptions that can give rise to misleading interpretations, and (3) robust evaluation of CA requires thorough consideration not only of active vasomotor function, but also the unique properties of the intracranial environment.

## What is the nature of the ‘challenge’?

The brain is one of the most metabolically active organs in the human body, accounting for 20 % of the body’s resting energy consumption despite weighing only 2 % of the total body mass (Clarke and Sokoloff 1999). This high energetic, and hence perfusion, demand means that the brain is highly susceptible to ischaemic injury. Conversely, overabundant cerebral blood flow (CBF) relative to metabolic need is also problematic in that this can result in the breakdown of the blood–brain barrier and permit the transudation of fluid into the interstitum and pericapillary astrocytes. Such changes have been shown to underlie the development of hyperperfusion syndromes, which are characterised by debilitating neurological sequelae including seizures, headaches, encephalopathy and stroke (van Mook et al. 2005). Stringent regulation of CBF is therefore pivotal to the maintenance of normal brain function.

**Fig. 1:**
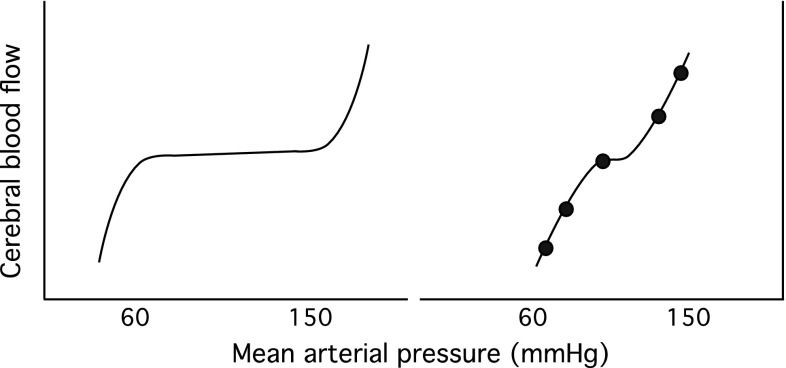
Stylised representation of the possible relationships between mean arterial pressure and cerebral blood flow. The *left panel* represents Lassen’s classic cerebral autoregulation curve. The *curve* on the *right panel* shows a more restricted autoregulatory plateau as indicated in recent studies (Tan 2012). Note that attempts to characterise static cerebral autoregulation using incremental drug infusion protocols may not reveal the plateau where blood pressure steps are greater than the width of the plateau (Lucas et al. 2010)

Cerebral autoregulation (CA) is thought to play a dominant role in CBF homeostasis. The classic description of CA is that CBF is maintained at a constant level across a wide range of mean arterial blood pressure (60–150 mmHg) (Lassen 1959). Based on steady-state CBF and blood pressure measurements made in patients afflicted with hypertensive and hypotensive disorders, this classical ‘model’ of CA has dominated our thinking on the relationship between blood pressure and cerebral perfusion (Fig. 1). However, the inadequacies of this concept began to emerge in the 1980s with the introduction of imaging techniques such as transcranical Doppler ultrasonography that provided relatively high fidelity recordings of CBF velocity (Aaslid et al. 1989). The realisation that CBF velocity (and by assumption CBF) was surprisingly dynamic at rest, and could alter markedly in response to dynamic changes in blood pressure gave rise to the concept of dynamic CA—a loosely defined term that refers to the regulation of cerebrovascular resistance against dynamic changes in blood pressure (Aaslid et al. 1989; Tiecks et al. 1995). The development of new methods for the characterisation of dynamic CA, and the practical application of these methods, has been a central focus of haemodynamic research over the last two decades (Aaslid et al. 1989; Ainslie et al. 2008; Bellapart et al. 2011; Carey et al. 2000; Panerai 2009b; Reinhard et al. 2003b).

**Fig. 2:**
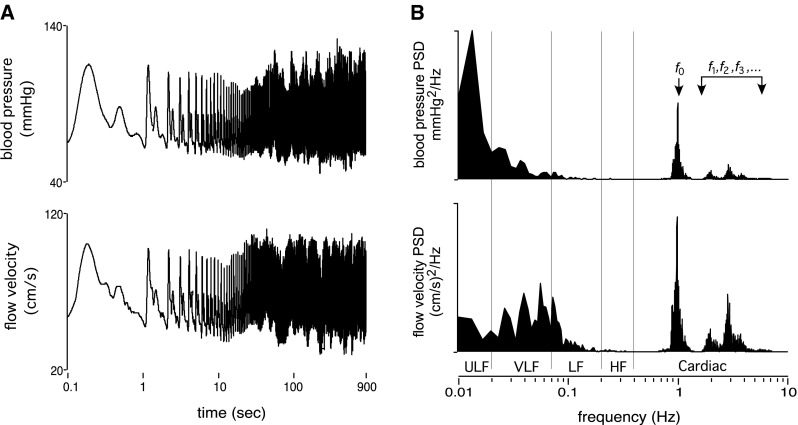
**a** Finger arterial blood pressure and middle cerebral blood flow velocity over 900 s (presented on log axes) for a human subject in the seated resting position. **b** The corresponding power spectrums, which decompose the time series signals into its various constituent component frequencies. Note that the fundamental frequency (*f*_o_) and its harmonics (*f*_1_, *f*_2_, *f*_3_) correspond to pulsations coincident with the pulse, whereas progressively lower frequency components reflect the longer term oscillations and trends in the time domain. *ULF* ultra low frequency, *VLF* very low frequency, *LF* low frequency, *HF* high frequency

**Fig. 3:**
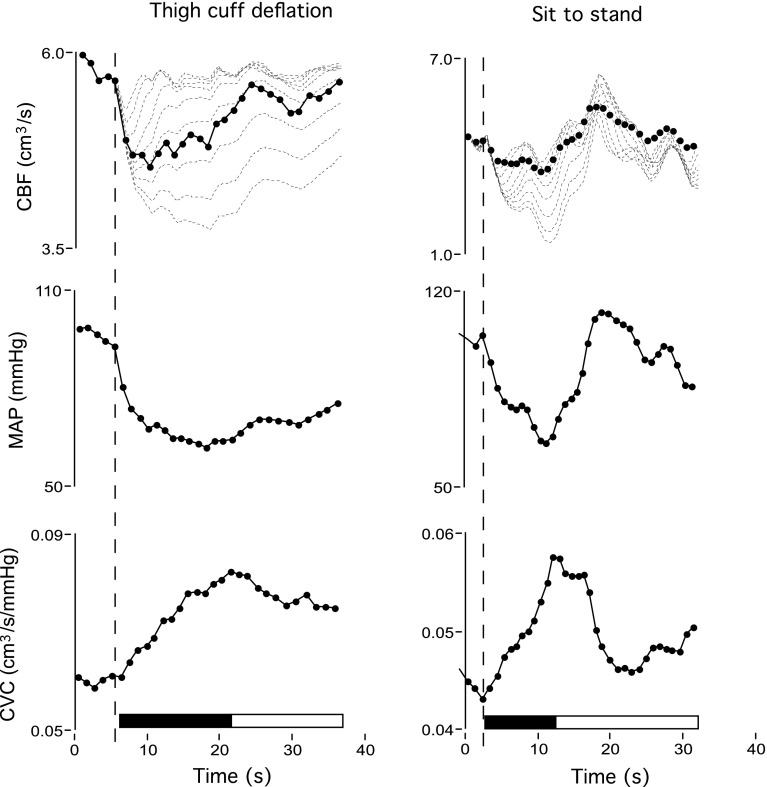
Mean cerebral blood flow (CBF), mean arterial blood pressure (MAP) and cerebrovascular conductance (CVC) estimated on a beat-to-beat basis for one subject during thigh cuff deflation (*left panel*) and during sit-to-stand (*right panel*). CBF estimates were made on the assumption that the middle cerebral artery radius is constant at 2 mm. *Vertical dashed* *line* indicates the time of thigh cuff deflation and stand. Both thigh deflation and standing from a sitting position elicited abrupt transient hypotension, although MAP recovery following cuff deflation occurred relatively slowly in this individual. Nevertheless, as indicated by the *dark horizontal bars*, CVC increased in a relatively linear fashion almost immediately (within one heart beat) following hypotension onset. The CBF *plot* shows the actual (*dots*) and CBF time course generated using Tieck’s autoregulatory model (*dashed lines*) (Tiecks et al. 1995). The rate of regulation index is typically calculated for the time period 1.0–3.5 s following the onset of thigh cuff deflation

Despite developments of new method and models of dynamic CA quantification, the translation of findings into clinical practice has been limited. On the one hand, old dogmatic paradigms are being maintained due to inadequate comprehension of the limitations of existing data that support established constructs. On the other hand, adoption of new concepts about CA has been hampered by the fact that much still remains uncertain about the fundamental physiology of CA regulation and quantification. As discussed in volume I of this thematic series, blood pressure is not a static variable but varies in magnitude across a range of time scales (Fig. 2). Observed over seconds, blood pressure resembles a complex pulsatile waveform with several characteristic features that are governed by intrinsic cardiovascular properties (e.g. ventricular pre-load, vascular compliance, blood density) and pulse wave reflection. Observed over a longer time scale, blood pressure exhibits naturally occurring trends and oscillations that span over minutes, hours, days and beyond. Superimposed on top of this natural level of oscillatory activity, one can expect non-rhythmic blood pressure surges and dips that occur either spontaneously, or as a consequence of physical activities such as exercise, straining, coughing, and changing body posture (Fig. 3). Thus, blood pressure is an amalgamated parameter that cannot be fully described in terms of simple numerical averages.

Do these rhythmic and non-rhythmic aspects of blood pressure challenge cerebral perfusion homeostasis? While research into the disease associations between blood pressure and cerebrovascular outcomes has primarily focused on average absolute blood pressure, clinical studies suggest that elevated short (minutes) and long-term (hours to days) blood pressure variability increases the likelihood of adverse cerebrovascular outcomes (Dawson et al. 2000; Ko et al. 2010; Rothwell 2010).

This leads us to the conceptual challenges of how we may appropriately probe the cerebral circulation to understand its fundamental processes. In clinical practice, many haemodynamics parameters (e.g. blood pressure, heart rate) are still frequently considered in the time domain summarised as averages (i.e. point estimates) even where continuous recordings are available. However, because blood pressure and blood flow exhibit dynamic fluctuations, it is beneficial to consider their relationships not only in terms of time-domain averages, but also in terms of different oscillatory components where progressively longer periodicities and trends correspond to progressively lower frequency components (Fig. 2). These are crucial distinctions because processes governing pressure and flow in the vasculature occur on different timescales and cannot be fully encapsulated by any single model of the circulation. While the pressure–flow relationship at ~0 Hz can be characterised using steady-state equations (e.g. Poisueille’s law), such equations may not necessarily apply to pressure–flow relationships at higher frequencies, and their inappropriate application can potentially give rise to misleading interpretations (see “What do these pressure–flow relationships”). Thus, it is important to discriminate between the pressure–flow phenomena that we observe from what we think explains the observations.

The focus of this paper is not to reiterate recent reviews in this area (Bor-Seng-Shu et al. 2012; Jordan and Powers 2012; Koller and Toth 2012). Rather, it is to provoke new lines of investigation by highlighting observations that challenge established paradigms about how blood pressure and CBF are fundamentally related. Because physiological inferences are only as valid as the assumptions inherent in our methodological approaches, we devote several sections to methodological background and considerations that are all too often ignored when interpreting data. Many controversies about how CBF is regulated against changes in blood pressure are likely the product of inadequate consideration of these methodological issues. This is followed by a brief consideration of the potential mechanisms that might be responsible for the cerebral autoregulatory response. Finally, we summarise the relationships between acute and chronic changes in blood pressure on CBF and highlight important areas for future investigation.

## What is the relationship between average blood pressure and average blood flow under steady-state conditions?

The haemodynamic determinants of average blood flow through an organ are typically understood through Poiseuille’s law, or the haemodynamic equivalent of Ohm’s law, known as Darcy’s law:

1 $$ Q = \frac{\Updelta P}{R} $$

In the context of the brain, ∆*P* is the cerebral perfusion pressure calculated from the difference between mean arterial pressure and the effective downstream pressure of the cerebral circulation, and R is the cerebrovascular resistance.

This is a well-established model of the circulation, but the question of how flow changes in response to changes in pressure across a wide range of steady states remains a subject of considerable controversy. In a review paper, Lassen constructed a plot of average pressure and flow from 7 studies involving 11 different patient groups with varying drug and pathology induced blood pressure levels (Lassen 1959). The plot revealed a plateau region wherein CBF appears to be constant across a relatively wide range of blood pressure (~60–150 mm Hg; Fig. 1). For this relationship to hold, increases and decreases in the average blood pressure must be accompanied, respectively, by increases and decreases in cerebrovascular resistance. In support of this notion, early observations of surface vessels of the brain have confirmed that both large and small pre-capillary cerebral arteries undergo ‘autoregulatory’ calibre adjustments in response to steady-state (and dynamic) increases and decreases in arterial blood pressure (Fog 1939a, b; Kontos et al. 1978). Reflex adjustments in vascular resistance to steady-state alterations in blood pressure are now commonly referred to as static CA.

Although Lassen’s diagram is widely cited as the ‘autoregulation curve’ describing the pressure–flow relations within an individual, it must be acknowledged that each data point on the curve derives from independent subjects, and therefore represents inter- (not intra-) individual relationships. Unfortunately, efforts to define the ‘correct’ static CA curve have been somewhat hindered by normal physiologic responses. For example, the baroreflex limits the blood pressure range that can be systematically explored without using vasoactive drugs, or shifting central blood volume. Further, changes in alveolar ventilation secondary to hypertension/hypotension can alter the partial pressure of arterial CO_2_ and therefore also alter CBF independently of CA. Although vasoactive drugs enable blood pressure to be systemically perturbed, their use can complicate the interpretation of results due to their potential effects on the tonus of both large and small arteries. For example, in healthy human volunteers stepwise increases and decreases in blood pressure (range 40–125 mmHg) using intravenous infusions of phenylephrine and nitroprusside are accompanied by more or less linear changes in middle cerebral artery velocity within the autoregulatory region (Lucas et al. 2010). However, it has been suggested that increases in middle cerebral artery blood velocity during steady-state phenylephrine infusions are not accompanied by measurable changes in internal carotid artery flow (Ogoh et al. 2011), raising the possibility that elevations in flow velocity may reflect an increase in arterial vascular tone, potentially of the insonated vessel itself.

Because it is difficult to distinguish CA from the vascular effects of drugs and other physiologic mechanisms, some studies have circumvented the use of drugs by studying the relationship between blood pressure and surrogates of cerebral perfusion averaged over relatively long durations using statistical techniques. Studies employing empirical (Czosnyka et al. 2001), piecewise (Brady et al. 2012), and projection pursuit regression (Tan 2012) under various experimental settings have all demonstrated pressure–flow curves resembling Lassen’s autoregulatory curve in that pressure-passive regions shoulder an intervening plateau (Fig. 1). However, studies are inconsistent in that the plateau regions are either relatively wide (>40 mmHg) (Brady et al. 2012) or demonstrably narrow (i.e. 10–15 mmHg) (Tan 2012).

So how effective is static CA? It seems that despite four decades of research, the answer to this question remains inconclusive and ultimately may well vary according to different experimental conditions. One pertinent consideration here is the purported role of cardiac output as a determinant of CBF independent of cerebral perfusion pressure (Ogoh et al. 2005). The apparent novelty of this concept arises from the fact that cardiac output is not typically considered as a determinant of flow according to Poiseuille’s law. This line of reasoning however neglects the fact that cardiac output is the net blood flow ejected out of the heart. Within the closed cardiovascular system, any interventions that alter cardiac output could also alter steady-state CBF via flow mediated changes in regional vascular resistance (Koller and Toth 2012). Most studies that have attempted to characterise static CA have not explicitly accounted for cardiac output as a determinant of CBF (separate to blood pressure and vascular resistance).

Precise characterisation of static CA has important clinical implications. Even with widespread availability of potentially suitable monitoring devices (e.g. Doppler ultrasound, near infrared spectroscopy), cerebral perfusion in clinical studies is still generally evaluated indirectly by measures of cerebral perfusion pressure or by internal jugular venous oxygen saturation. Because average blood pressure is a major clinical indicator and widely considered in clinical practice, clarifications of concepts such as the lower and upper limits of static CA are required if these concepts are to serve the management of haemodynamically compromised individuals. Here matters are further complicated by the fact that anaesthesia is often used in conjunction with surgery in many critical scenarios and many anaesthetic agents have direct cerebral vasodilatory effects that can alter the fundamental characteristics of static CA (Reinsfelt et al. 2003, 2011). This concern underpins previous suggestions that different static CA curves may apply under different physiological states (Drummond 1997).

## What is the dynamic relationship between blood pressure and blood flow?

Whereas static CA refers to the relationship between average cerebral perfusion pressure and average blood flow under steady-state conditions, ‘dynamic’ CA refers to the vascular responses to higher frequency components of steady-state spontaneous blood pressure, or to dynamic changes in blood pressure (such as those driven by altered body posture). Although this terminology only came into popular use in the 1980s, recognition that cerebral arterioles are capable of responding dynamically over relatively short time scales to blood pressure transients dates back to animal experiments showing that pial pre-capillary arterioles under direct observation undergo autoregulatory changes in vessel calibre with dynamic changes in blood pressure (Fog 1939a, b).

Several important factors were subsequently found to modulate the development and characteristics of this vascular response. First, significant vessel calibre changes typically did not occur until blood pressure had reached a certain threshold (MAP ~90 mmHg) (Kontos et al. 1978). Below this threshold, progressive reductions in blood pressure gave rise to progressive increases in vessel calibre until the point of maximal dilatation. Second, vascular dilatation to hypotension was determined in part by the rate of change in blood pressure. Dilatation occurred more quickly and with a shorter latency (~5 s) if the blood pressure reduction was large and abrupt. In contrast, dilatation developed more slowly in response to slower changes in blood pressure such as those caused by inferior vena caval obstruction, haemorrhage, or infusion of vasodilators. Third, the duration of hypotension did not seem to be critical for the development of vasodilatation. Provided that the magnitude of hypotension was sufficient, even brief period (2–3 s) of hypotension elicited vasodilatation. In contrast to the vasodilator response, comparatively little is known about the dynamics of the vasoconstrictor response. Most data report exclusively the effects of drug-induced increases in blood pressure, which typically occur slowly and are accompanied by less pronounced vasoconstrictor response. Whether the aforementioned factors that influence the vasodilatation response are also important in the generation of the vasoconstrictor response remains unclear due to the technical difficulties associated with generating controlled elevations in blood pressure.

While studies based on the direct visualisation of cerebral vessel calibre proved the existence of dynamic CA, they do not present a practical approach to characterise autoregulatory function in conscious humans. Nor do they quantify the extent to which flow is successfully buffered against transient changes in blood pressure. The answer to this question required simultaneous high fidelity recordings of blood pressure and CBF. In a classic study, Aaslid and colleagues used transcranial Doppler to characterise the CBF velocity responses to abrupt release of inflated thigh occlusion cuffs (Aaslid et al. 1989). Cuff deflation induced sudden stepwise drops in arterial blood pressure that remained depressed for approximately 5–7 s before gradual restoration to baseline levels over the following 10–20 s via cardiac and vascular baroreflex-mediated increases in blood pressure. Simultaneous recordings of middle cerebral arterial blood velocity showed a similar response pattern except that following the initial stepwise drop, flow velocity recovered more rapidly than blood pressure (Fig. 3). Comparable patterns of pressure and flow velocity recovery have been described in response to transient hypotension induced with orthostatic stimuli, such as standing from sitting position (Sorond et al. 2009), or bolus injections of vasoactive drugs (Chan et al. 2011; Tzeng et al. 2010b). These time-domain relationships confirmed the conceptual inadequacies of Lassen’s CA curve by showing that cerebral flow stabilising mechanisms achieve relative, rather than absolute, CBF buffering. However, these findings also suggest that factors other than blood pressure contribute to effective flow stabilisation.

**Fig. 4:**
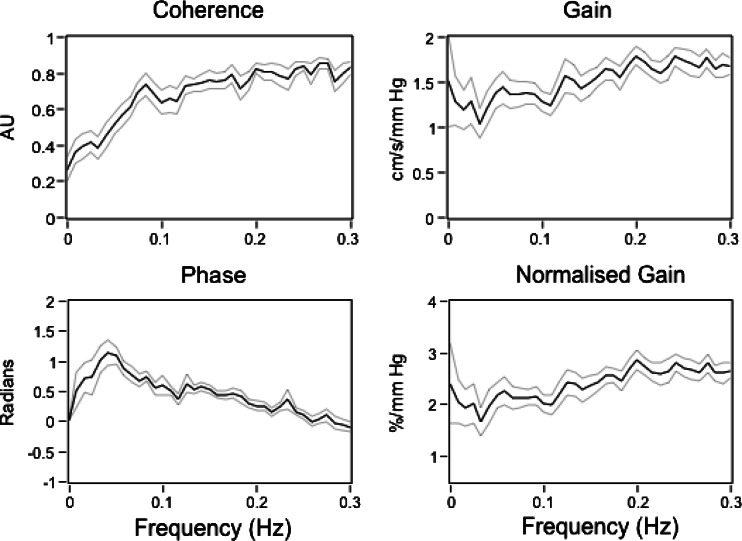
Transfer function coherence, phase, gain and normalised gain for 105 healthy individuals (mean age 26 ± 7 years) in the supine resting position. Data adapted from (Tzeng et al. 2012). *AU* arbitrary units. Values are mean ± SE

The concepts of dynamic flow buffering were further reinforced by studies using analytical techniques such as spectral and linear transfer function analysis in the frequency domain (Giller 1990). By decomposing pressure and blood flow velocity recordings into their various oscillatory components, transfer functions can define the relationships between blood pressure (input) and flow velocity (output) in terms of their linear statistical dependence (coherence), relative magnitudes (gain), and timing (phase) as a function of the frequency component of interest (Zhang et al. 1998) (Fig. 4). Studies have consistently shown that for frequencies below ~0.07 Hz, the cross-spectral coherence is generally low (i.e. <0.5), indicating that less than 50 % of the flow velocity variance can be explained by changes in blood pressure. Between 0.07 and 0.20 Hz, there is a pattern of progressively increasing coherence and gain indicating that higher frequency blood pressure components are more linearly related to flow velocity than lower frequency components, and that the ratio between them increases. Also, within this range, phase is consistently positive, indicating that flow paradoxically leads pressure but the phase lead progressively falls towards zero when approaching 0.2 Hz. Between 0.2 and 0.5 Hz, coherence and gain are elevated and the phase remains around zero, indicating that pressure and flow are linear and synchronously related. This cross-spectral pattern has been confirmed in a number of studies, and has been likened to a ‘high pass filter’ wherein lower frequency blood pressure components are more effectively filtered allowing only the transmission of higher frequency fluctuations (Hamner et al. 2004; Reinhard et al. 2003b; Tzeng et al. 2012; Zhang et al. 2002). For summary purposes, the transfer function is often averaged across each of the three different frequency bands, on the assumption that each band reflects a different aspect of cerebrovascular physiology.

## What do these pressure–flow relationships tell us about CBF control?

Hitherto, the relationships between blood pressure and flow described above have been explained on the basis of conceptual paradigms that emphasise the role of CA over other potential mechanisms. As shown in Fig. 3, because the flow velocity reduction following transient hypotension is proportionately less pronounced than the blood pressure decrease, cerebral vascular (flow/pressure) calculated on a beat-to-beat basis, also increased. Such increases in beat-to-beat cerebral vascular conductance are believed to reflect a linearly evolving vascular dilation response, and are the basis for metrics such as the rate of regulation index (Aaslid et al. 1989) and Tiecks autoregulatory index (Tiecks et al. 1995). Likewise, the transfer function characteristics within the 0.02–0.4 Hz range have been attributed to the capacity for cerebral arterioles to dilate and constrict dynamically in response to increases and decreases in the blood pressure (Deegan et al. 2009, 2011; Hamner et al. 2004; Reinhard et al. 2003a; Zhang et al. 1998). This assumption underlies the use of transfer function coherence (Giller 1990), gain (Hamner et al. 2010) and phase (Immink et al. 2004), and related techniques such as impulse response function (Zhang et al. 1998) as metrics of dynamic CA.

**Fig. 5:**
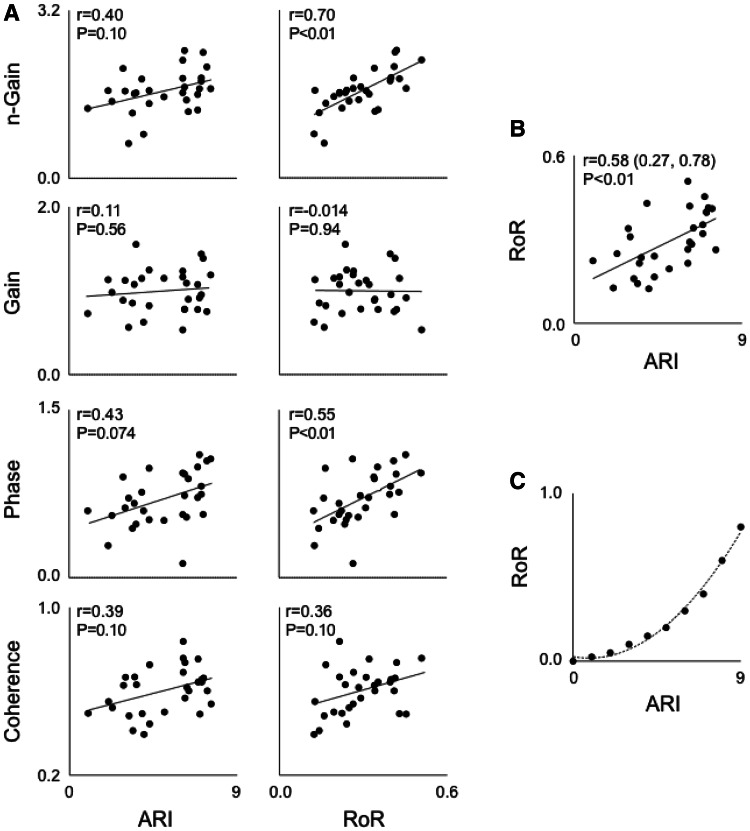
*Scatter plots* showing the relationships between various metrics of dynamic cerebral autoregulation. **a** The relationships between autoregulatory index (ARI) and rate of regulation index (RoR) derived from thigh cuff deflations test and metrics derived from spontaneous transfer function analysis in the 0.07–0.2 Hz range (*n* = 29). ARI showed no clear relationships with any transfer function metric. RoR was positively related to phase and *n*-gain. The latter association does not support the conventional interpretation of these metrics, given that RoR and *n*-gain should be inversely related. **b** The relationships between RoR and ARI, which are metrics derived from the same pressure–flow recordings. Note the high degree of data dispersion compared to **c**, which shows the theoretically predicted relationship between RoR and ARI. All data are adapted from (Tzeng et al. 2012) and (Tiecks et al. 1995)

**Fig. 6:**
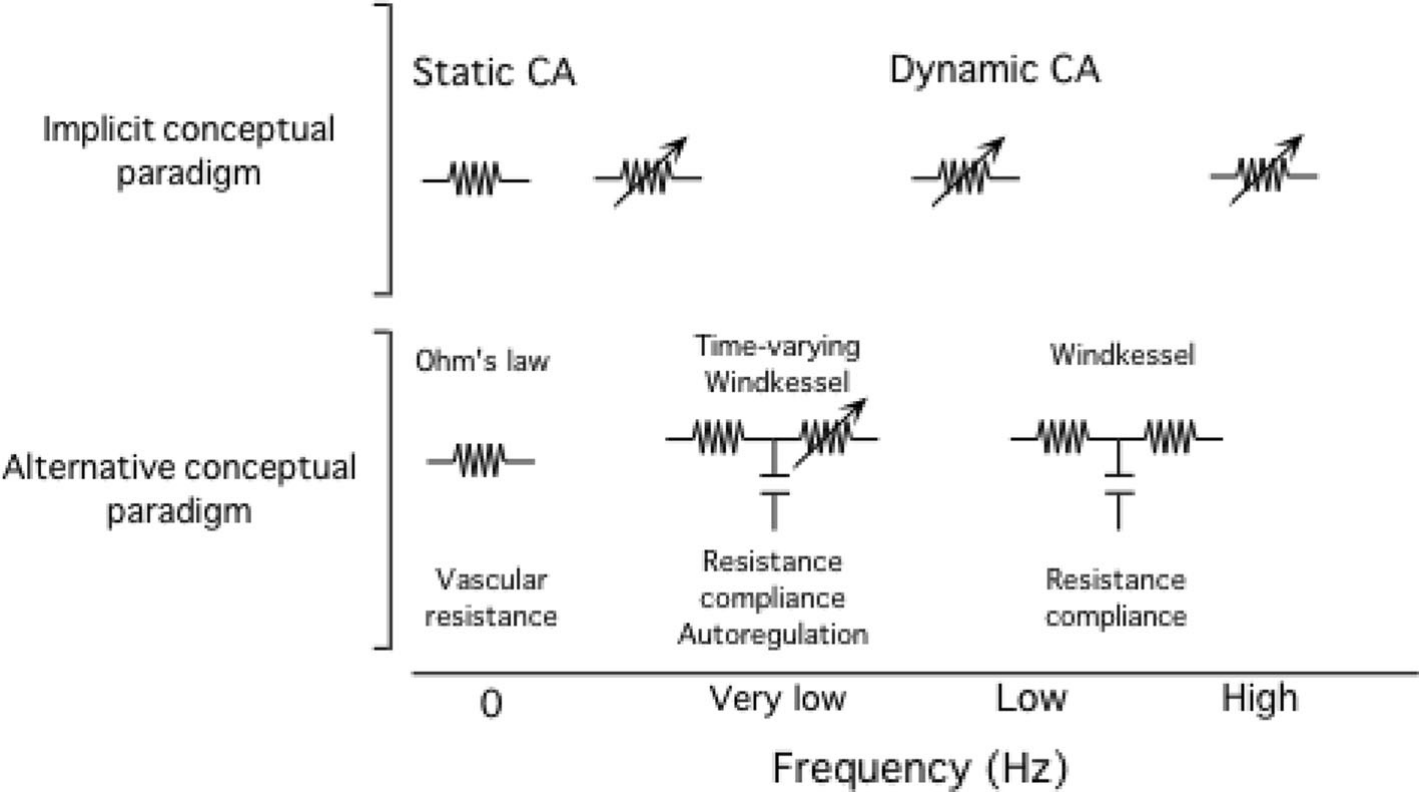
Schematic diagram illustrating how different conceptual paradigms can influence data interpretation. The *top panel* shows the popular implicit paradigm that assumes cerebral autoregulation (CA) as the principal determinant of cerebral pressure–flow velocity relationships. The *bottom panel* shows one potential alternative paradigm that accounts for inherent vascular properties such as resistance and compliance in addition to CA. Such alternative models require further experimental validation. Potential influences due to other processes such as neurovascular coupling and partial pressure of arterial are not shown

The implicit assumption that these metrics quantitatively reflect dynamic CA is challenged by clinical studies showing that dynamic CA do not always provide congruent results (Panerai 2008, 2009a). For example, one study found that phase reductions in MCA stenosis were not associated with changes in gain (Haubrich et al. 2003). In contrast, another showed that increases in gain in autonomic failure were not accompanied by phase changes (Blaber et al. 1997). Recently we compared the relationships between several commonly applied metrics of dynamic CA and found that few exhibit statistical associations with each other to support a common functional basis (Fig. 5) (Tzeng et al. 2012). Furthermore, we found that alterations in the partial pressure of end-tidal CO_2_ influenced transfer function coherence, gain, and phase at different frequencies to different extents, and with different levels of consistency across individuals. This raises the challenge and, as-yet, unanswered question of which metric, or combination of metrics, one should use to assess dynamic CA? Answering this question requires a more thorough consideration of the factors that may modulate dynamic pressure–flow relationships and, potentially, a critical revision of conceptual paradigms that are currently applied to their interpretation (Fig. 6).

## What factors can potentially influence our interpretation of pressure–flow relationships?

Alone or in combination, technical/experimental factors and differences in conceptual paradigm(s) may influence our interpretation of pressure–flow relationships.

### Technical/experimental factors

An important technical consideration that applies to studies employing transcranial Doppler is that blood velocity measurements reflect volumetric blood flow only if the cross-sectional area of the insonated vessel remains constant. Any changes in vascular diameter will introduce errors in the estimation of flow (Holland et al. 1996). While such diameter changes may not occur in humans at the level of the MCA where flow velocity recordings are often taken (Serrador et al. 2000), abrupt hypotension can elicit passive reductions in vascular diameter that can be up to 10 % of the baseline value (Kontos et al. 1978). Considering that even a 3 % diameter change (a level not readily detectable by cerebral magnetic resonance imaging) could eliminate the changes in calculated vascular resistance observed shortly after a blood pressure transient (Kontos 1989), such changes can greatly affect the validity of metrics such as the RoR index (Aaslid et al. 1989; Ogoh et al. 2008, 2010; Tzeng et al. 2010a, b). Therefore, researchers employing transcranial Doppler need to carefully consider the potential for experimental interventions to alter the calibre of the insonated vessels.

Another methodological consideration relates to the frequent estimation of cerebral perfusion pressure using indirect methods. Theoretically, cerebral perfusion pressure is calculated from the difference between mean central arterial pressure and the effective downstream pressure of the cerebral circulation. Because the transmural pressure in distal cerebral veins is very close to zero, the intracranial pressure can become the principal determinant of the postcapillary venous outflow pressure in a manner that is similar to a Starling resistor (Greenfield and Tindall 1965). However, it has been suggested that the effective downstream pressure may be determined by a critical closing pressure that is the sum of the intracranial pressure and the active tension produced by vascular smooth muscle contraction at the arteriolar level (Dewey et al. 1974), and is theoretically the pressure at which flow ceases. Because critical closing pressure can be as high as 30 mmHg in the supine position (Aaslid et al. 2003), or even higher in pathological intracranial hypertension (Alperin et al. 2012), the common practice of ignoring effective downstream pressure can lead to marked overestimation of true cerebral perfusion pressure.

### Conceptual paradigms

Inferences of vascular properties and behaviour based on different conceptual frameworks can give rise to different interpretations of the same data. Given that dynamic CA is an inherently non-linear process, it is important to consider the appropriateness of using linear steady-state models to draw inferences on dynamic behaviour. The utility of Poiseuille’s law, for example, arises from the fact that we can use it to infer vascular resistance or conductance. Blood vessels are assumed to be constricting if resistance is increasing. If resistance is decreasing, we infer blood vessels are dilating. This interpretation belies the fact that vascular resistance is a specific construct that describes the relationship between average pressure and flow under steady state. The common practice of calculating vascular resistance as the ratio of mean pressure and mean flow on a beat-to-beat basis during non-steady-state conditions in a compliant vascular bed ignores that varying amounts of blood can be stored in the compliance ‘reservoir’ due to the blood acceleration or deceleration (Essler et al. 1999; Shim et al. 1994; Toorop et al. 1987).

The following question arises: is it appropriate to dismiss vascular properties such as compliance in the cerebral circulation? The prevailing paradigm is that cerebral arteries are stiff vessels and therefore compliance plays little to no role in buffering the passage of blood flow through the cranial compartment (Aaslid et al. 1991). Proponents of this ‘rigid-tube’ model cite as evidence of the apparently short pulse transit times between cerebral arterial and venous pulse waves (Aaslid et al. 1991), that cerebral arteries possess relatively little elastin in the tunica media and adventitia (Hayashi et al. 1980; Nagasawa et al. 1979), and these arteries exhibit high resistance to axial deformation (Monson et al. 2003, 2005). Moreover, given that intracranial contents within the rigid skull (blood, brain tissue, and cerebral spinal fluid) are nearly incompressible, constraints on cerebral vascular compliance by the global intracranial compartment compliance are to be expected (Hu et al. 2006). On the other hand, fresh human cerebral arteries subjected to multi-axial deformation show anisotropic stress-stretch behaviour with considerably less resistance to deformation around the circumference than in the axial direction (Monson et al. 2008). Cerebral pressure–flow relationships characterised across a range of time scales using Windkessel models that incorporate a compliance reservoir are able to generate flow waveforms that fit well with actual flow recordings under a variety of dynamic situations (Chan et al. 2011; Gommer et al. 2012; Tzeng et al. 2011; Zhang et al. 2009). These findings allude to the presence of capacitive-type flow that is driven by the rate of change in blood pressure rather than absolute blood pressure per se.

In addition to elastic vascular properties, there is also preliminary evidence that changes in beat-to-beat stroke volume can affect some metrics of dynamic CA. For example, total cardiac autonomic blockade using metoprolol and glycopyrrolate leads to a reduction in the rate of regulation index (Ogoh et al. 2010). Parameters such as stroke volume or heart rate are not routinely incorporated in the assessment of CA and its importance remains unclear (Deegan et al. 2010; Ogoh et al. 2010). Other factors that are well recognised in their influences on CBF but are not yet routinely accounted for in analysis of dynamic CA include neurovascular coupling (Spronck et al. 2012), flow/sheer stress interactions (Peterson et al. 2011), and spontaneous fluctuations in the partial pressure of arterial CO_2_ (Marmarelis et al. 2013).

## Can there be multiple interpretations for the same observation?

Considering physiological processes other than CA can have dramatic implications for data interpretation in both the time and frequency domain alike. For example, assuming a purely resistive paradigm, the beat-to-beat decreases in the cerebral vascular resistance following thigh cuff deflation (Aaslid et al. 1989) or standing from a sitting position (Tzeng et al. 2010b) would indicate that vascular dilation evolves linearly within one to two cardiac cycles of hypotension onset. For individuals with an average heart rate of ~60 beats per min, this translates to a time delay of 1–2 s (Fig. 3), which is considerably shorter than the mean delay of 5–10 s between the onset of acute hypotension and onset of cerebral arteriolar vasodilatation under direct observation (Kontos et al. 1978). In contrast, beat-to-beat cerebral vascular resistance estimated using a 3-element Windkessel model shows a bi-phasic response to transient hypotension (Olufsen et al. 2002). Immediately upon standing, the reduction in blood pressure is accompanied by (1) an initial increase in peripheral vascular resistance, either as part of baroreflex-mediated sympathetic activation or secondary to passive changes in vessel calibre, and (2) a secondary decrease in resistance approximately 10 s after the initial onset of hypotension (Olufsen et al. 2002).

Steady-state vascular properties are equally important when interpreting data presented in the frequency domain. The pattern of rising gain, falling phase, and the phase lead of flow on pressure going from 0.02 to 0.4 Hz is frequently attributed to CA (Hughson et al. 2001). However, such qualitative features can be expected of a linear 3-element Windkessel model that does not incorporate any active autoregulation (Gommer et al. 2012). Thus, although transfer function phase or gain may reflect dynamic CA, both metrics could also partly reflect steady-state vascular properties such as compliance and resistance (Zhang et al. 2009). A relevant additional consideration here is that linear transfer functions characterise time-invariant systems and therefore yield robust phase and gain estimates only if the linearity/coherence assumption is satisfactorily meet. Because dynamic CA is an active time-varying response, its characterisation may not be possible when pressure and flow are linearly related, such as in the LF and HF range under spontaneous conditions (Tzeng et al. 2012), or when the blood pressure is active-driven (e.g. cyclic squat–stand manoeuvres, oscillatory lower body negative pressure) (Tzeng et al. 2011). These considerations underpin recent suggestions that steady-state cerebrovascular resistance and/or vascular compliance modulate the dynamic pressure–flow relationship at the low and high frequencies, while dynamic CA is likely to be dominant at the very low frequencies (Chan et al. 2011; Tzeng et al. 2011; Zhang et al. 2009).

Given that a gold-standard method for dynamic CA quantification has yet to be established, our specific methodological choices should be made in cognisance of the objectives of the research. Currently, studies frequently ascribe physiological meaning to data on the a priori assumption that the chosen metric faithfully reflects dynamic CA. The pitfall of this practice is that conceptual biases can be generated and perpetuated that then erroneously ‘confirm’ the validity of a particular paradigm. We advocate the use of existing methodologies primarily as tools for characterising cerebral haemodynamic relationships. Studies should consider a range of possible explanations for observable relationships that may include dynamic CA as well as other potential mechanisms.

## What physiological mechanisms are responsible for the cerebral autoregulatory response?

With particular focus on human studies, this section will discuss the physiological mechanisms thought to underpin CA. Several theories have been advanced supporting the role of the endothelium, extravascular nerves, endogenous circulating catecholamines, and the vascular smooth muscle itself. For comprehensive coverage, readers are referred to recent topical reviews (Peterson et al. 2011; Toda et al. 2009). Here, we will focus on the findings from studies in healthy humans, which can be broadly defined into three domains: endothelial related regulation (e.g. nitric oxide), neurogenic regulation (e.g. sympathetic activity), and intrinsic myogenic control. Rather than recapitulating the conclusions of the individual studies, here we attempt to highlight the potential complexities surrounding data interpretations.

### Nitric oxide

Using the thigh cuff deflation method, White et al. (2000) showed that patients treated with l-NMMA exhibited a ~17 % reduction in autoregulatory index, which was unchanged in control patients treated with an equivalent presser dose of noradrenaline. Given the reduction in autoregulatory index occurred despite an increase in vascular tone secondary to nitric oxide synthase inhibition, it was argued that the nitric oxide partly mediated the vasodilatory response to transient hypotension. In contrast, using transfer function analysis, Zhang et al. (2004) concluded that the tonic production of nitric oxide does not alter dynamic CA, given that l-NMMA inhibition did not alter transfer function phase and gain relationships between pressure and flow velocity in the LF range. The discrepancies observed between these studies are consistent with the lack of metric convergence common between studies employing different experimental and analytical techniques.

### Neurogenic factors

In contrast to the abundance of studies in animals (reviewed in Sandor 1999), relatively few studies have directly examined the possibility of autonomic neurovascular control in humans. It has been shown that ganglionic blockade with Trimethaphan increased transfer function gain and reduced phase, suggesting that dynamic CA might be impaired (Zhang et al. 2002). However, ganglion blockade disrupts all autonomic neural modulation and therefore does not allow the isolation of sympathetic and cholinergic effects. Unfortunately, studies that have sought to specifically identify the role of the sympathetic and cholinergic system in dynamic CA have provided inconsistent results. In healthy volunteers treated with Prazosin, the rate of regulation index has been shown to decrease by ~58 % (Ogoh et al. 2008). In contrast, the rate of regulation index does not differ between controls and patients with infarction of the dorsal–lateral medulla oblongata affecting central sympathetic pathways (Gierthmuhlen et al. 2011). More recently, healthy human volunteers treated with phentolamine and glycopyrrolate have been shown to enhance transfer function gain at frequencies >0.06 Hz. These findings suggest there are sympathetic (Hamner et al. 2010) as well as cholinergic (Hamner et al. 2012) influences on the cerebrovasculature. However, given that pressure and flow were highly coherent in both studies due to the use of oscillatory lower body negative pressure, it remains unclear to extent that observed gain changes reflect altered dynamic CA. Furthermore, phentolamine altered baseline end-tidal CO_2_ partial pressure, which introduces an important confounder that could have been accounted for through end-tidal CO_2_ partial pressure clamping (Willie et al. 2012).

### Myogenic factors

The origins of vascular responses to changes in blood pressure are thought to arise in part from the vascular smooth muscle itself, independent of endothelial or neuronal input. This myogenic concept essentially proposes that stretching of vascular smooth muscles activates stretch-sensitive ion channels (Bayliss 1902), which in turn initiate membrane depolarisation through non-selective cation channels, resulting in the influx of Ca^2+^ through voltage-gated Ca^2+^ channels, and consequently smooth muscle contraction (Hill et al. 2006; Jackson 2000). To date, no studies have satisfactorily assessed the relative importance of the myogenic response in maintaining CBF in vivo because the cerebrovascular bed cannot be easily isolated from all other exogenous physiologic influences (e.g. neural, metabolic). Further attempts to fully abolish the myogenic response is complicated by the blood–brain barrier, which limits the types of Ca^2+^ channel blockers that can used to reliably investigate the role of Ca^2+^ channels in the integrated regulation of CBF.

Nevertheless, few studies have examined the impact of l-type calcium channel blockade on dynamic CA in humans. Using the thigh cuff deflation method, Endoh et al. (2000) reported a 37 % reduction in autoregulatory index after Nicardipine treatment in anaesthetised patients during gynaecologic or orthopaedic surgery. More recently, we studied the effects of Ca^2+^ channel blockade on cerebral pressure–flow velocity relationships during oscillatory lower body negative pressure (Tzeng et al. 2011). As demonstrated previously (Hamner et al. 2004), active augmentation of blood pressure fluctuations at 0.05 Hz enhanced cross-spectral coherence consistent with enhanced linearity between pressure and flow. Under these conditions, Ca^2+^ channel blockade reduced phase but did not alter gain. To determine whether the phase changes were due to altered CA or changes in cerebral Windkessel properties, the data were fitted to a 2-element Windkessel model that partitions net MCA flow into: (1) MAP-driven resistive flow through resistance vessels, (2) capacitive flow through compliant vessels driven by the rate of change in MAP, and (3) a residual component that reflects non-linear processes including CA. We found that Ca^2+^ channel blockade enhanced the resistive flow component without altering capacitive flow, and that steady-state vasodilation explained the vast majority (~90 %) of middle cerebral artery blood velocity variance. These findings provided the first quantitative illustration of how transfer function metrics do not necessarily reflect dynamic CA. Interestingly, Ca^2+^ channel blockade reduced middle cerebral artery blood velocity in all subjects even though Nimodipine is a potent cerebral vasodilator (Canova et al. 2012; Haws et al. 1983). These seemingly paradoxical changes caused by middle cerebral artery dilation (Canova et al. 2012) may explain why Ca^2+^ channel blockade did not alter transfer function gain.

## How do acute and chronic blood pressure challenges influence CBF?

It is beyond the scope of the current review to describe in depth every physiological condition that may influence BP and CBF. Here we selectively focus on the following relevant topics:

### Transient hypertension, hypotension and CBF

It has been suggested that the magnitude of CBF responses to transient blood pressure changes varies depending on whether blood pressure is rising or falling. In patients with head injury, dynamic CA (quantified using Tieck’s autoregulatory index) maybe more adept at compensating for transient hypertension than for hypotension (Aaslid et al. 2007). In healthy volunteers, increases in middle CBF velocity associated with phenylephrine-induced increases in blood pressure are comparatively smaller in magnitude than flow velocity reductions due to nitroprusside or orthostatically induced decreases in blood pressure. Asymmetric pressure–flow relationships may reflect an evolutionary adaptation for protecting the cerebral circulation against arterial pressure surges (Cassaglia et al. 2008). Animal data indicate that cerebral sympathetic nerves are activated during acute hypertension, but not hypotension (Cassaglia et al. 2009). Furthermore, transfer function gain and coherence between middle CBF velocity and MAP oscillations induced with oscillatory lower body negative pressure are enhanced following alpha-adrenergic blockade (Hamner et al. 2009). The rise in coherence may be interpreted as an increase in linearity between MAP and MCAv_mean_ relations due to the blockade of cerebral sympathetic vasoconstriction. However, it is important to recognise that human pial arteries are sparsely innervated and are insensitive to topical noradrenaline (Bevan et al. 1998). Therefore, it remains possible that asymmetric cerebral pressure–flow relations may relate to differences in intrinsic myogenic responses to hypotension vs. hypertension.

### Exercise-induced BP changes and CBF

Exercise poses a relevant challenge for CA. For example, during cycling exercise, MAP and systolic blood pressure increase by 20–30 %, combined with a slight decrease or no change in diastolic blood pressure (Rowell 1974). These changes during exercise are often more pronounced with ageing (Marsden et al. 2012). During static exercise (e.g. weightlifting), supraphysiological elevations in blood pressure up to 450/380 mmHg have been reported in elite power athletes. As such, dynamic changes in systemic blood pressure, such as those seen during both resistance and dynamic exercise, present a substantial challenge to cerebral homeostasis. However, despite progressive elevations in systolic and MAP during exercise, it is known that CBF only increases by 15–25 % until 70 % of maximal oxygen uptake. Thereafter, CBF declines towards or below baseline during hyperventilatory-induced hypocapnia, despite progressive increases in perfusion pressure (Ogoh and Ainslie 2009). During resistance exercise, however, evidence indicates that CBF decreases rather than increases likely due to the intense Valsalva and elevations in intracranial pressure (Dickerman et al. 2000). It therefore appears that the cerebral circulation is extremely well adapted to protect the brain from over perfusion due to elevations in blood pressure with exercise.

### Chronic hypertension, hypotension and CBF

Both chronic hypertension and hypotension are generally associated with paradoxical reductions in CBF. With chronic hypertension, long-term structural changes in small cerebral vessels, brain atrophy, and altered blood–brain barrier permeability are known to occur (Cohen 2007). Such effects of hypertension on the cerebrovasculature are likely the result of reductions in CBF (Beason-Held et al. 2007; Cohen 2007; Waldstein et al. 2010). Earlier studies have shown that with chronic hypertension, the so-called static autoregulatory curve shifts rightward placing patients at risk of impaired ability to tolerate hypotension (Lassen 1974). However, evidence for the impaired capacity to cope with transient hypotension has not been established to our knowledge. Nevertheless, long-term treatment of hypertension may partially reverse this rightward shift. In a recent trial on hypertensive (>150 mmHg) elderly (>70 years), it was reported that intensive lowering of BP to <130/80 versus the usual clinical target of <140/80 produced increased CBF as determined by 3 T arterial spin labelling MRI (Tryambake et al. 2013).

With chronic hypotension with and without conditions of low cardiac output, the related brain cerebral hypoperfusion has shown to cause cognitive deficits in attention and memory (Duschek et al. 2005). In addition, more recent studies propose that these deficits develop from brain hypoperfusion and can lead to Alzheimer’s disease (Qiu et al. 2003; Waldstein et al. 2005). However, the link between and cognitive decline has been generally ignored in clinical practice. This in likely to be due, in part, to the belief that low systemic blood pressure does not cause brain dysfunction because compensatory CA prevents brain hypoperfusion from being activated (Duschek and Schandry 2007). However, studies have confirmed, particularly in the elderly, that CA does not necessarily protect the brain from chronic low blood pressure and low cardiac output, an outcome that can result in CBF insufficiency and its accompanying consequences (Duschek and Schandry 2007; Kennelly and Collins 2012; Nilsson et al. 2007; Qiu et al. 2003). In summary, both chronic hypotension and hypertension can lead to significant reductions in CBF. Chronically reduced CBF is clearly linked to cognitive decline (Goldstein and Reivich 1991), post-stroke dementia (Firbank et al. 2011) and late-life depression (Colloby et al. 2012). Thus, the fundamental challenge of BP changes on CBF can have very long-term consequences.

## Conclusions and future directions

In this review, we have deliberately highlighted important but often ignored discrepancies in the literature surrounding the characterisation of CA. These discrepancies point to a need to formulate new studies and measurement strategies that overcome those methodological limitations. Such work is crucial because our ability to understand how the cerebral circulation responds to blood pressure challenges rests on robust methods of assessment. Given that dynamic CA is frequently characterised indirectly by inference, close scrutiny needs to be applied to the methodological and conceptual assumptions we adopt. Some of these are outlined in “Can there be multiple interpretations for the same observation?”. Furthermore, because CA is a non-linear mechanism that interacts dynamically with other physiologic processes (e.g. partial pressure of arterial CO_2_, neurovascular coupling), ongoing analytical innovations that enable multivariate quantification of both linear as well as non-linear properties of the cerebral circulation are needed.

Finally, it is important to reiterate that the challenges discussed in this review do not preclude the use of existing techniques in clinical investigations. Whilst the elucidation of underlying control mechanisms is a principal focus, characterisation of the endpoint parameters that regulatory systems purportedly control (e.g. blood pressure and CBF variability) may yield equally important information. Indeed, many endpoint parameters, including the time-honoured average blood pressure, have proven clinical utility despite the incomplete understanding of their underlying physiology. Considering there is ready access to a range of technologies for quantifying blood pressure and CBF variability, clinical research that is focused on risk factor identification and monitoring can and must continue despite the physiological complexities we have sought to highlight.
